# First Report of Swim Bladder-Associated Microbiota in Rainbow Trout (*Oncorhynchus mykiss*)

**DOI:** 10.1264/jsme2.ME17071e

**Published:** 2018-12-28

**Authors:** Alejandro Villasante, Carolina Ramirez, Natalia Catalán, Jaime Romero

**Affiliations:** 1 Laboratorio de Biotecnología, Instituto de Nutrición y Tecnología de los Alimentos (INTA) Universidad de Chile Avda. El Líbano 5524, Santiago Chile; 2 Laboratorio de Biotecnología, Unidad de Alimentos, Instituto de Nutrición y Tecnología de los Alimentos (INTA) Universidad de Chile Avda. El Líbano 5524, Santiago Chile

Volume 32, No. 4, Page 386–389, 2017

<Page 1, left column>
  L4: Ref. 9 and 17 should be replaced with 10 and 19, respectively.
  L10: Ref. 7 and 8 should be replaced with 8 and 9, respectively.
  L14: “cost-efficient buoyancy-controlling” should be replaced by “energy cost-efficient buoyancy-controlling”.
  L15: Ref. 23 and 26 should be replaced with 26 and 30, respectively.
  L18: Ref. 24 and 27 should be replaced with 28 and 31, respectively.
  L21: Ref. 14 should be replaced with 15.
  L24: Ref. 24 should be replaced with 28.
  L28: Ref. 25 should be replaced with 17 and 29.
  L33: Ref. 18 should be replaced with 20.
<Page 1, right column>
  L6: Ref. 3 and 20 should be replaced with 4 and 23, respectively.
  L21: “every d” should be replaced by “every day”.
  L25: Ref. 13 should be replaced with 14.
<Page 2, left column>
  L4: Ref. 12 should be replaced with 13.
  L9: Ref. 12 should be replaced with 14.
  L10: Ref. 12 should be replaced with 14.
  L32: Ref. 10 should be replaced with 11.
<Page 3, left column>
  L5: Ref. should be (16, 17, 18).
  L20: Ref. should be (6, 22).
  L25–L26: should be (16, 18, 21).
  L-27: Ref. 16 should be replaced with 27.
<Page 3, right column>
  L3: Ref. should be (5, 24).
  L5–L6: Ref. should be (7, 25).
  L29: Ref. 11 should be replaced with 12.
  L29: Add “in mammals, including human” after “a unique steady-state microbiota”.
  L33: Ref. should be (4, 23).

<References>

The references after ref. 6 should be changed as follows:

7. Franco, R., and J.A. Cidlowski. 2009. Apoptosis and glutathione: beyond an antioxidant. Cell Death Differ. 16:1303–1314.
8. Gratacap, R.L., J.F. Rawls, and R.T. Wheeler. 2013. Mucosal candidiasis elicits NF-κB activation, proinflammatory gene expression and localized neutrophilia in zebrafish. Dis. Models & Mech. 6:1260–70.
9. Gratacap, R.L., and R.T. Wheeler. 2014. Utilization of zebrafish for intravital study of eukaryotic pathogen–host interactions. Dev. Comp. Immunol. 46:108–115.
10. Green, T., R. Smullen, and A. Barnes. 2013. Dietary soybean protein concentrate-induced intestinal disorder in marine farmed Atlantic salmon, *Salmo salar* is associated with alterations in gut microbiota. Vet. Microbiol. 166:286–292.
11. Holben, W.E., P. Williams, M.A. Gilbert, M. Saarinen, L.K. Sarkilahti, and J.H. Apajalahti. 2002. Phylogenetic analysis of intestinal microflora indicates a novel Mycoplasma phylotype in farmed and wild salmon. Microb. Ecol. 44:175–185.
12. Marsland, B.J., and E.S. Gollwitzer. 2014. Host-microorganism interactions in lung diseases. Nat. Rev. Immunol. 14:827–835.
13. Navarrete, P., F. Magne, P. Mardones, M. Riveros, R. Opazo, A. Suau, P. Pochart, and J. Romero. 2010. Molecular analysis of intestinal microbiota of rainbow trout (*Oncorhynchus mykiss*). FEMS Microbiol. Ecol. 71:148–156.
14. Navarrete, P., F. Magne, C. Araneda, P. Fuentes, L. Barros, R. Opazo, R. Espejo, and J. Romero. 2012. PCR-TTGE analysis of 16S rRNA from rainbow trout (*Oncorhynchus mykiss*) gut microbiota reveals host-specific communities of active bacteria. PLoS One 7:e31335.
15. Nilsson, S. 1983. Autonomic Nerve Function in the Vertebrates. Springer-Verlag, New York.
16. Pelster, B. 1995. Metabolism of the swim bladder tissue, p. 101–118. In P.W. Hochachka and T.P. Mommsen (ed.), Biochemistry and Molecular Biology of Fishes, vol. 4. Elsevier Science, Amsterdam.
17. Pelster, B. 2001. The generation of hyperbaric oxygen tensions in fish. News Physiol. Sci., 16:287–291.
18. Pelster, B., M. Giacomin, C.M. Wood, and A.L. Val. 2016. Improved ROS defense in the swimbladder of a facultative air-breathing erythrinid fish, jeju, compared to a non-air-breathing close relative, traira. J. Comp. Physiol. B 186:615–624.
19. Ringø, E., Z. Zhou, J.L.G. Vecino, et al. 2016. Effect of dietary components on the gut microbiota of aquatic animals. A never-ending story? Aquacult. Nutr. 22:219–282.
20. Roberts, J.R. 2012. Fish Pathology. 4th ed. Blackwell Publishing Ltd. Oxford, Garsington.
21. Schneebauer, G., R. Hanel, and B. Pelster. 2016. *Anguillicola crassus* impairs the silvering-related enhancements of the ROS defense capacity in swimbladder tissue of the European eel (*Anguilla anguilla*). J. Comp. Physiol. B 186:1–11.
22. Schumann, P., N. Weiss, and E. Stackebrandt. 2001. Reclassification of *Cellulomonas cellulans* (Stackebrandt and Keddie 1986) as *Cellulosimicrobium cellulans* gen. nov., comb. nov. Int. J. Syst. Evol. Microbiol. 51:1007–1010.
23. Segal, L.N., and M.J. Blaser. 2014. A brave new world: the lung microbiota in an era of change. Ann. Am. Thorac. Soc. 11:S21–S27.
24. Shelly, C., and M.D. Lu. 2014. Glutathione synthesis. Biochim. Biophys. Acta 1830:3143–3153.
25. Smirnova, G.V., and O.N. Oktyabrsky. 2005. Glutathione in bacteria. Biochemistry (Moscow). 70:1199–1211.
26. Smith, F.M., and R.P. Croll. 2011. Autonomic control of the swimbladder. Auton. Neurosci. 165:140–148.
27. Veldkamp, H., G. van der Berg, and L.P.T.M. Zevenhuizen. 1963. Glutamic acid production by *Arthrobacter globiformis*. Antonie Van Leeuwenhoek 29:35–51.
28. Winata, C.L., S. Korzh, I. Kondrychyn, V. Korzh, and Z. Gong. 2010. The role of vasculature and blood circulation in zebrafish swimbladder development. BMC Dev. Biol. 10:3.
29. Woolley, L.D., and J.G. Qin. 2010. Swimbladder inflation and its implication to the culture of marine finfish larvae. Rev. Aquacult. 2:181–190.
30. Zaccone, D., M. Sengar, E.R. Lauriano, S. Pergolizzi, F. Macri’, L. Salpietro, A. Favaloro, L. Satora, K. Dabrowski, and G. Zaccone. 2012. Morphology and innervation of the teleost physostome swim bladders and their functional evolution in non-teleostean lineages. Acta Histochem. 114:763–772.
31. Zheng, W., Z. Wang, J.E. Collins, R.M. Andrews, D. Stemple, and Z. Gong. 2011. Comparative transcriptome analyses indicate molecular homology of zebrafish swimbladder and mammalian lung. PLoS One 6:e24019.

